# Human Papillomavirus Downregulates the Expression of IFITM1 and RIPK3 to Escape from IFNγ- and TNFα-Mediated Antiproliferative Effects and Necroptosis

**DOI:** 10.3389/fimmu.2016.00496

**Published:** 2016-11-22

**Authors:** Wenbo Ma, Bart Tummers, Edith M.G. van Esch, Renske Goedemans, Cornelis J.M. Melief, Craig Meyers, Judith M. Boer, Sjoerd H. van der Burg

**Affiliations:** ^1^Department of Medical Oncology, Leiden University Medical Center, Leiden, Netherlands; ^2^Department of Gynaecology, Leiden University Medical Center, Leiden, Netherlands; ^3^Department of Immunohematology and Blood Transfusion, Leiden University Medical Center, Leiden, Netherlands; ^4^Department of Microbiology and Immunology, The Pennsylvania State University College of Medicine, Hershey, PA, USA; ^5^Human Genetics, Leiden University Medical Center, Leiden, Netherlands

**Keywords:** HPV, infection, immune escape, Th1 cytokines

## Abstract

The clearance of a high-risk human papillomavirus (hrHPV) infection takes time and requires the local presence of a strong type 1 cytokine T cell response, suggesting that hrHPV has evolved mechanisms to resist this immune attack. Using an unique system for non, newly, and persistent hrHPV infection, we show that hrHPV infection renders keratinocytes (KCs) resistant to the antiproliferative- and necroptosis-inducing effects of IFNγ and TNFα. HrHPV-impaired necroptosis was associated with the upregulation of several methyltransferases, including EZH2, and the downregulation of RIPK3 expression. Restoration of RIPK3 expression using the global histone methyltransferase inhibitor 3-deazaneplanocin increased necroptosis in hrHPV-positive KCs. Simultaneously, hrHPV effectively inhibited IFNγ/TNFα-mediated arrest of cell growth at the S-phase by downregulating *IFITM1* already at 48 h after hrHPV infection, followed by an impaired increase in the expression of the antiproliferative gene *RARRES1* and a decrease of the proliferative gene *PCNA*. Knockdown of *IFITM1* in uninfected KCs confirmed its role on *RARRES1* and its antiproliferative effects. Thus, our study reveals how hrHPV deregulates two pathways involved in cell death and growth regulation to withstand immune-mediated control of hrHPV-infected cells.

## Introduction

High-risk human papillomaviruses (hrHPVs) infect undifferentiated keratinocytes (KCs) of squamous epithelia. Persistent infections may lead to cancers of the anogenital region as well as of the head and neck (1). In order to establish a persistent and productive infection, hrHPV requires access to the undifferentiated KCs that make up the epithelial basal layer and have the capacity to divide (2). hrHPV infections can persist despite viral activity in KCs, indicating that HPV has developed mechanisms to evade or suppress the innate and/or adaptive immune response of the host. Indeed, hrHPV utilizes its viral proteins and exploits cellular proteins to interfere with signaling of innate immune pathways, potentially postponing the activation of an adaptive immune response (3). HPV may attenuate immune signaling at different levels in the STAT (4–7), IRF, and NFκB pathways (8–14), and has also been shown to impair IFNγ and TNFα signaling (15). Nevertheless, T cells will become activated and migrate to infected sites. Studies in healthy individuals, immunosuppressed patients, and in patients with spontaneously or vaccine-induced regressions revealed an important role for a strong type 1 (IFNγ and TNFα)-associated HPV early antigen-specific T cell response in the control of HPV-infected lesions (16). However, even vaccines that boost viral Th1 immunity during chronic infection are only partially successful (17–19) with a positive clinical outcome only in patients with a very strong Th1 response (19, 20), suggesting that hrHPV may have found ways to resist the effector cytokines of the adaptive immune system.

IFNγ is a pleiotropic cytokine that affects immune regulation, immune surveillance, inflammation, and tumor suppression and has antiviral as well as antiproliferative properties. Binding of IFNγ to the IFNγ receptor (IFNγR) leads to JAK1/2-mediated STAT1 phosphorylation, dimerization, and nuclear translocation that results in interferon-stimulated gene (ISG) expression (21). IFNγ may also induce necroptotic cell death by the JAK1/STAT1-dependent activation of proteins, encoded by interferon-stimulated genes that drive various aspects of the RIPK1–RIPK3 necrosome complex assembly (22), including the RNA-responsive protein kinase PKR, which then interacts with RIPK1 to trigger necroptosis (23). TNFα also regulates cell survival, apoptosis, or necroptosis *via* an intricate network of signals that operate downstream of TNF receptor 1 (TNFR1). Binding of TNF to TNFR1 leads to NFκB activation through cIAP-mediated ubiquitylation of RIPK1. Under circumstances where RIPK1 is deubiquitylated, it can associate with FADD to recruit and homodimerize caspase-8 leading to the induction of apoptosis. RIPK1 can also bind and activate RIPK3 after which MLKL is activated and necroptosis ensues. cFLIP_L_ is expressed upon NFκB signaling (as *CFLAR*), heterodimerizes with caspase-8 on FADD, and, as such, prevents apoptosis by abrogating full caspase-8 activation and necroptosis by disrupting the interaction between RIPK1 and RIPK3 (24).

IFNγ and TNFα are known to synergize in the suppression of KC proliferation (25). IFNγ induces growth arrest and differentiation (26, 27). TNFα also induces growth arrest, but there are conflicting data concerning its capacity to induce cell death of primary KCs (25, 28). Exposure of KCs to both IFNγ and TNFα potently stimulates the production of nitric oxide synthase (iNOS) to induce the upregulation of FasL, Fas receptor activation, and subsequent caspase-mediated apoptosis of KCs (29), an effect to which differentiated KCs are especially sensitive (30). However, the ability of HPV-infected undifferentiated KCs to resist the effects of IFNγ and/or TNFα on proliferation as well as the underlying mechanisms are not well understood. In this study, we evaluated the influence of hrHPV on the IFNγ- and TNFα-mediated cell growth inhibition and cell death induction of undifferentiated KCs by functional and biochemical assays. We utilized a system that resembles the natural infection with HPV as closely as possible, comprising of primary KCs that stably maintain the hrHPV genome as episomes and were shown to undergo the entire differentiation-dependent HPV life cycle in organotypic raft cultures, non-infected primary KC cultures, and primary KCs newly infected with authentic HPV16 virions (13), to show that hrHPV presence renders KCs more resistant to both necroptosis and the antiproliferative effects instigated by IFNγ and TNFα and reveal the biological mechanisms responsible.

## Materials and Methods

### Ethics Statement

The use of discarded human foreskin, cervical, and vaginal KC tissues to develop cell lines for these studies was approved by the institutional review board at the Pennsylvania State University College of Medicine and by the institutional review board at Pinnacle Health Hospitals. The Medical Ethical Committee of the Leiden University Medical Center approved the human tissue sections (healthy foreskin, healthy cervix, and HPV16- or 18-positive cervical neoplasia) used for staining. All sections and cell lines were derived from discarded tissues and de-identified, therefore no informed consent was necessary.

### Cell Culture

Primary cultures of human epithelial KCs were established from foreskin, vaginal, vulva, and cervical tissues as previously described (31) and grown in keratinocyte serum-free medium (EpiLife^®^ Medium, with 60 μM calcium supplemented with HKGS kit, Invitrogen, Breda, The Netherlands). KCs stably maintaining the full episomal HPV genome following electroporation (HPV-positive KCs) were grown in monolayer culture using E medium in the presence of mitomycin C-treated (Sigma-Aldrich) J2 3T3 feeder cells (32, 33) for two passages and were then adapted to EpiLife^®^ Medium for one passage before experimentation. J2 3T3 mouse fibroblasts were cultured in Iscove’s modified Dulbecco’s medium supplemented with 8% fetal bovine serum, 2 mM l-glutamine, and 1% penicillin–streptomycin (complete IMDM medium) (Gibco-BRL, Invitrogen).

### Reagents

Recombinant human TNFα (Rhtnf-a, Invivogen/bioconnect, France), Recombinant Human Interferonγ (11343536, Immunotools, Germany), BV-6 (S7597, Selleckchem, Netherlands), Pan Caspase Inhibitor Z-VAD-FMK (FMK001, R&Dsystems, USA), 3-Deazaneplanocin A (DZNep) hydrochloride (A8182, Apexbt, Netherlands), necrostatin (Nec)-1s (2263-1, Biovision, CA, USA), Cycloheximide (CHX) solution (C4859, SIGMA, Netherlands), and GSK503 (S7804, Selleckchem, Netherlands).

### Analysis of IFNGR1, TNFR1, and TNFR2 Cell Surface Expression

The expression of the receptors for the cytokines IFNγ and TNFα was analyzed by flow cytometry after staining of the cells with the antibodies (1:10 diluted) against IFNGRα (Mouse anti-human CD119-PE, clone GIR-94, BD Biosciences, Breda, The Netherlands), TNFR1 (mouse anti-human CD120a-PE, clone 16803, R&D systems, Abingdon, UK), or TNFR2 (mouse anti-human CD120b-PE, clone MR2.1, Invitrogen Life Technologies, Bleiswijk, The Netherlands). Briefly, cells were transferred into wells of V-bottom 96-wells plate, washed with ice-cold PBS + 0.5% BSA, and incubated for 10 min on ice with ice-cold PBS (B. Braun, Melsungen, Germany) + 0.5% bovine serum albumin (Sigma-Aldrich) + 10% fetal calf serum (PAA Laboratories, Lelystad, The Netherlands). Then, the cells were washed again and incubated for 30 min on ice in the dark with the antibody indicated. Following one wash step, the cells were fixed in 1% paraformaldehyde before they were acquired by BD Fortessa with BD FACSDiv software version 6.2 and data analyzed using FlowJo version 10.0.7 (Treestar, Olten, Switzerland).

### HPV16 Knockdown in HPV16-Positive KCs and Infection of Undifferentiated Keratinocytes

HPV16-positive KCs were transfected with 50 nM Control or HPV16 E2 siRNA as previously described (13). Primary basal layer human foreskin KCs were infected with native HPV16 at MOI 100 as previously described (13). Cells were washed and harvested, and target gene expression was assayed by RT-qPCR.

### IFITM1 Knockdown in Undifferentiated KCs

shRNA’s were obtained from the MISSION TRC-library of Sigma-Aldrich (Zwijndrecht, The Netherlands). The MISSION shRNA clones are sequence-verified shRNA lentiviral plasmids (pLKO.1-puro) provided as frozen bacterial glycerol stocks (Luria Broth, carbenicillin at 100 μg/ml, and 10% glycerol) in *E. coli* for propagation and downstream purification of the shRNA clones. pLKO.1 contains the puromycin selection marker for transient or stable transfection. The construct against interferon-induced transmembrane protein 1 (IFITM1) (NM_003641) was TRCN0000057499: CCGGCCTCATGACCATTGGATTCATCTCGAGATGAATCCAATGGTCATGAGGTTTTTG and the control was: SHC004 (MISSION TRC2-pLKO puro TurboGFP shRNA Control vector): CCGGCGTGATCTTCACCGACAAGATCTCGAGATCTTGTCGGTGAAGATCACGTTTTT. KCs at ~60% confluency were transduced with lentivirus at MOI 5–10 over night, after which medium was replaced. At least 72 h post-transduction, cells were harvested, washed, and plated as indicated and allowed to attach overnight. Cells were stimulated as indicated and assayed accordingly.

### RNA Expression Analyses

The micro array data (14) is accessible in the Gene Expression Omnibus database (accession number GSE54181). Plots were generated using the webtool R2: microarray analysis and visualization platform (http://r2.amc.nl).

Total RNA was isolated using the NucleoSpin RNA II kit (Machery-Nagel, Leiden, The Netherlands) according to the manufacturer’s instructions. Total RNA (0.5–1.0 μg) was reverse transcribed using the SuperScript III First Strand synthesis system from Invitrogen. TaqMan PCR was performed using TaqMan Universal PCR Master Mix and pre-designed, pre-optimized primers and probe mix for IFITM1, GLB1, BCL-2, Bax, RARRES1, PCNA, and GAPDH (Applied Biosystems, Foster City, USA). Threshold cycle numbers (Ct) were determined using the CFX PCR System (Bio-Rad, Veenendaal, The Netherlands), and the relative quantities of cDNA per sample were calculated using the ΔΔCt method using GAPDH as the calibrator gene.

### Western Blot Analysis

Polypeptides were resolved by SDS-polyacrylamide gel electrophoresis (SDS-PAGE) and transferred to a nitrocellulose membrane (Bio-Rad, Veenendaal, The Netherlands). Immunodetection was achieved with primary antibodies against TRAF2 (#4724s, Cell Signaling Technology (CST), Leiden, Netherlands), cIAP1 (#7065p, CST), cIAP2 (#3130s, CST), XIAP (#14334, CST), RIPK1 (#3493, CST), cFLIP (#3210, CST), caspase-8 (#9746, CST), cleaved caspase-8 (#9496s, CST), FADD (#2782, CST), RIPK3 (#13526, CST), MLKL (#14993s, CST), phospho-MLKL (phospho S358; ab187091, Abcam, Cambridge, UK), EZH2 (612667, BD Biosciences, The Netherlands), IFITM1 (PA5-20989, Thermo Scientific, Netherlands), Trimethyl-Histone H3 (Lys27) (07-449, Merk Millipore), STAT1 (#9172, CST), phospho-STAT1 (Tyr701, #9167, CST), β-actin (A5316, Sigma-Aldrich, Germany), and HRP-coupled anti-mouse (#7076s, CST) and HRP-coupled anti-rabbit (#7074s, CST) secondary antibodies. Chemoluminescence reagent (#170-5060, Bio-Rad, Germany) was used as substrate, and signal was scanned using the Chemidoc and accompanying Software (Image Lab Software Version 5.2.1, Bio-Rad).

### Proliferation Assay

Keratinocytes, hrHPV + KCs, control shRNA-expressing KCs, or IFITM1 shRNA-expressing KCs were seeded 5000 cell/well in 96-well plates and allowed to attach overnight. Cells were cultured in presence of indicated concentrations of IFNγ and/or TNFα in 150 μl for 96 h. 15 μl/well MTT (3-(4,5-dimethylthiazol-2-yl)-2,3-diphenyl-2H-tetrazolum bromide) stock solution (5 mg/ml in 0.1M PBS) was added for 3 h. When the purple formazan precipitate was clearly visible under the microscope, bright light pictures were made using an Olympus IX51 inverse fluorescence microscope (Olympus, Zoeterwoude, The Netherlands). Images were captured by ColorView II Peltier-cooled charge-coupled device camera (Olympus) and archived using Cell^^^F software (Olympus).

### Cellular DNA Content Analysis

The CyQuant-NF assay (C35006, ThermoFisher Scientific, USA), which measures cellular DNA content *via* fluorescent dye binding, was used to quantify the cell number in cultures treated with increasing doses of IFNγ. Briefly, control shRNA-expressing KCs or IFITM1 shRNA-expressing KCs were seeded 500 cell/well in 96-well plates and allowed to attach overnight. Cells were cultured in presence of indicated concentrations of IFNγ, in triplicate wells, for 96 h and then processed according to the protocol for adherent cells provided by the manufacturer. The fluorescence intensity detected is a measure for the number of cells present in the wells (34).

### Cell Cycle Analysis of Keratinocytes

Following the treatment of KCs with 250 IU/ml IFNγ for 48 h, the cells were fixed in 70% ethanol at 4°C overnight. The fixed cells were washed with cold PBS and subsequently incubated for 30 min with 10 μg/ml RNase (#R6513, Sigma-Aldrich) and 10 μg/ml propidium iodide (P4170, Sigma-Aldrich) staining. Cell cycle was detected by flow cytometry (BD Accuri™ C6, BD biosciences, The Netherlands) and analyzed using FlowJo v10.0.8.

### SYTOX Green Dead Cell Assay

Replicate cultures of cells were plated in 6-well tissue culture plates. Following the indicated treatments, all adhering and floating cells were collected with TrypLE™ Express Enzyme (12604-021, Thermofisher). The cells were washed with HBSS (14025-092, Thermofisher) once and then incubated with 5 μM SYTOX^®^ Green Nucleic Acid Stain (S7020, Thermofisher) in absence or presence of 1 μg/ml DAPI (D9542, Sigma-Aldrich) at 20°C for 30 min. The cells were washed with HBSS and mixed with VECTASHIELD antifade mounting medium (Vectorlabs H-1000). A 30-μl cell suspension was added to a glass section slide and examined by fluorescence microscopy. Non-DAPI stained cells were detected by flow cytometry (BD Accuri™ C6) and analyzed using FlowJo v10.0.8.

### Statistics

Statistical analysis was performed using GraphPad Prism version 6.02. *P* values were determined using Welch-corrected unpaired *t*-tests or one-way ANOVA Tukey’s multiple comparisons test. Ns: no significance. *P < 0.05, **P < 0.01, ***P < 0.001, ****P < 0.0001.

## Results

### HPV-Infected KCs Have an Altered Expression of Genes Related to Necroptosis and Proliferation in Response to IFNγ and TNFα Stimulation

**Figure 1:**
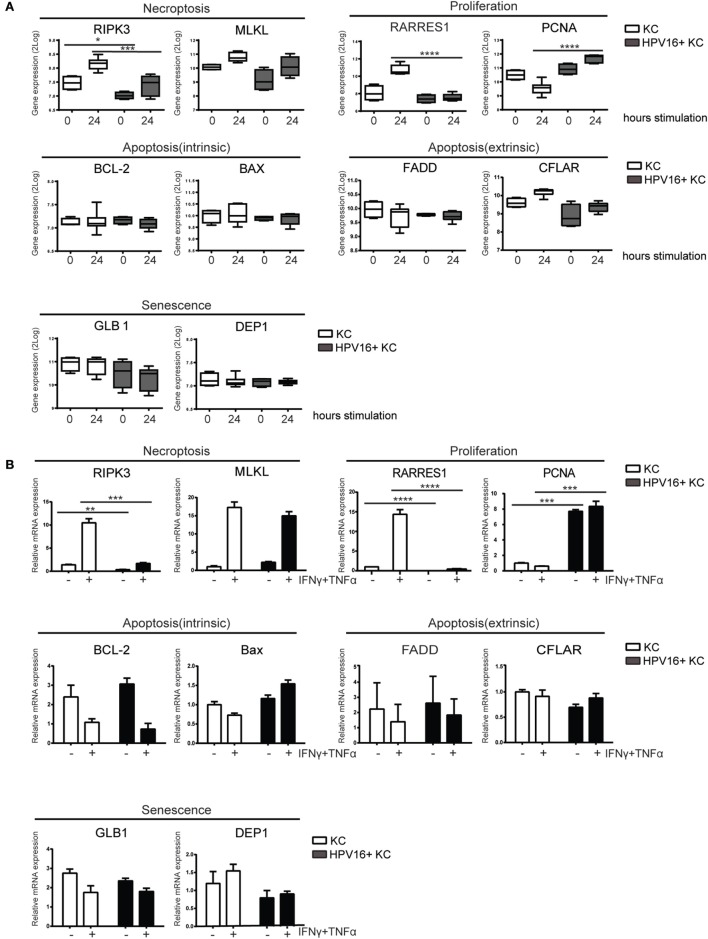
**HPV-infected KCs display an altered expression of genes related to necroptosis and proliferation**. **(A)** Microarray gene expression values for *RIPK3, MLKL, RARRES1, PCNA, BCL-2, BAX, FADD, CFLAR, GLB1*, and *DEP1* of four independent uninfected KCs and four independent hrHPV + KCs, stimulated with IFNγ for 0 or 24 h, represented in a box plot. The box represents the 25th and 75th percentiles, the median is indicated with a horizontal line within the box, and the whiskers represent the minimum and maximum. ***P < 0.001 and ****P < 0.0001. **(B)** RT-qPCR of *RIPK3, MLKL, RARRES1, PCNA, BCL-2, BAX, FADD, CFLAR, GLB1*, and *DEP1* in uninfected KCs and HPV16 + KCs following treatment with 50 IU/ml IFNγ and 30 ng/ml TNFα for 24 h. Gene expression was normalized against *GAPDH* mRNA levels and standardized against the non-stimulated uninfected KCs. Similar results were observed in two independent experiments. ****P < 0.0001.

We previously reported our validated microarray in which four independent uninfected KC and four independent hrHPV-infected KC cultures were stimulated with control or IFNγ (14, 31). Analysis of marker genes in this array for necroptosis (*RIPK3, MLKL*), proliferation (*RARRES1, PCNA*), intrinsic apoptosis (*BCL-2, BAX*), extrinsic apoptosis (*FADD, CFLAR*), and senescence (*GLB1, DEP1*) revealed that hrHPV infection was specifically associated with changes in the genes associated with necroptosis and proliferation (Figure 1A). *RIPK3*, a crucial regulator of necroptosis, and its downstream partner *MLKL* (35) were both downregulated in IFNγ-stimulated hrHPV-infected KCs (Figure 1A). Furthermore, the increase in *RARRES1*, a marker for antiproliferation (36, 37), and the decrease in *PCNA*, a marker of proliferation, observed in KCs treated with IFNγ for 24 h was not seen in hrHPV-positive KCs where expression of these genes remained almost unaltered (Figure 1A). These data suggest that hrHPV-infected KCs can resist the growth regulatory effects of IFNγ and/or the combination of IFNγ and TNFα. Uninfected KCs and hrHPV-infected KCs express the IFNγ receptor 1 (IFNGR1), and the TNFα receptors 1 and 2 (TNFR1 and TNFR2) at the cell surface enabling them to respond to these cytokines (Figure S1A in Supplementary Material). When these cells were seeded into 96-well plates and treated for 4 days with increasing doses of IFNγ and/or TNFα, the growth of uninfected KCs was greatly affected by IFNγ in a dose-dependent manner while hrHPV-positive KCs were much more resistant to growth inhibition and still able to expand to a confluent cell layer (Figure S1B in Supplementary Material). TNFα in itself appeared not to affect the growth of uninfected or HPV-infected KCs, but, when combined with IFNγ, it exaggerated the reduction in cell density (Figure S1B in Supplementary Material). To confirm these results, KCs were harvested after IFNγ/TNFα stimulation, and the gene expression of the previously indicated markers indicative for proliferation, senescence, apoptosis, and necroptosis were determined by RT-qPCR (Figure 1B). The qPCR showed that *RIPK3* is lower in hrHPV-infected KCs, both at the basal level and after treatment, while the effect on *MLKL* was less pronounced. Moreover, the increase in *RARRES1* and downregulation of *PCNA* was much lower in treated hrHPV-infected KC than in non-infected KCs (Figure 1B). Similar to the microarray, the marker genes for intrinsic and extrinsic apoptosis as well as senescence did not overtly differ between KCs and hrHPV-positive KCs. Together, our results show that specifically the necroptosis-associated gene *RIPK3* and the antiproliferative gene *RARRES1* were expressed lower in hrHPV-positive KCs. This suggests that the maintained proliferation of hrHPV-infected undifferentiated KCs during IFNγ and/or TNFα treatments is associated with a resistance to cell death at the level of necroptosis and by resistance to proliferation arrest, but less likely to be regulated at the level of senescence or apoptosis.

### HPV Suppresses Necroptosis by Downregulating RIPK3 Expression

**Figure 2:**
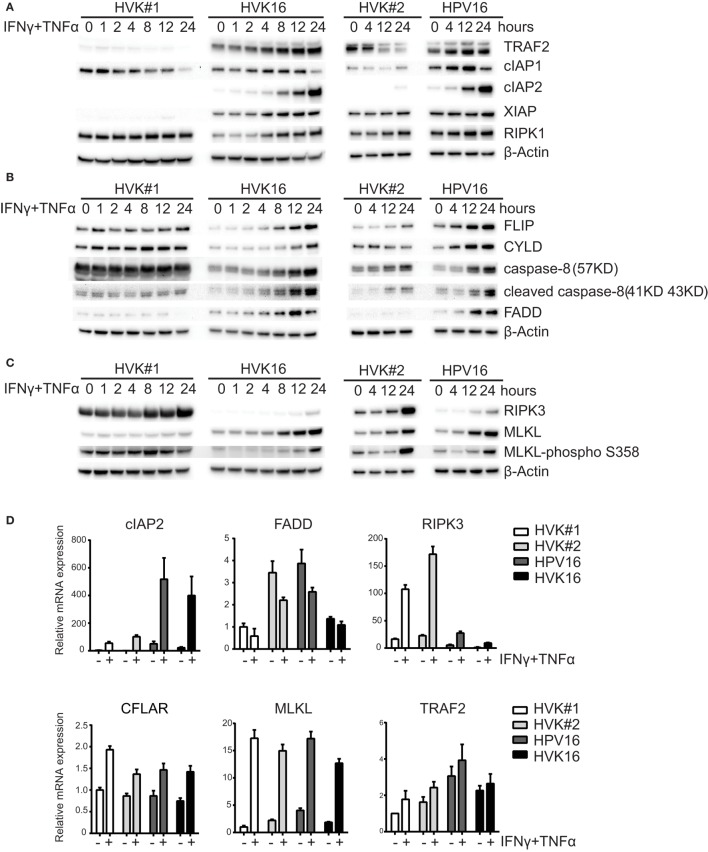

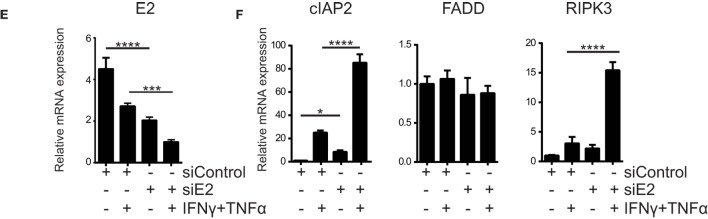
**HPV suppresses the expression of RIPK3**. **(A–C)** Two independent uninfected KC cultures (HVK#1, HVK#2) and two HPV16 + KC cultures (HVK16, HPV16) were stimulated with 50 IU/ml IFNγ and 30 ng/ml TNFα for the indicated times. The protein expression levels of **(A)** TRAF2, cIAP1, cIAP2, XIAP, RIP1; **(B)** FLIP, CYLD, caspase-8 (57 kD), cleaved caspase-8 (41 kD, 43 kD), FADD; and **(C)** RIPK3, MLKL and MLKL-phospho S358 as detected by Western blotting (WB) in whole cell extracts. β-actin served as loading control. **(D)** Two independent uninfected KC cultures (HVK#1, HVK#2) and two HPV16 + KC cultures (HVK16, HPV16) were stimulated with 50 IU/ml IFNγ and 30 ng/ml TNFα for 24 h after which the expression levels of *cIAP2, FADD, RIPK3, CFLAR, MLKL*, and *TRAF2* were determined by RT-qPCR. Gene expression was normalized against *GAPDH* mRNA levels and standardized against the non-stimulated HVK#1. Similar results were observed in two independent experiments. **(E)** HPV16 *E2* expression in HPV + KCs transfected with control siRNA (siControl) or siRNA targeting HPV16 E2 (siE2) stimulated with or without 50 IU/ml IFNγ and 30 ng/ml TNFα for 24 h. *E2* expression was analyzed by RT-qPCR. Gene expression was normalized against *GAPDH* mRNA levels and standardized against siControl. Similar results were observed in three independent experiments. ***P < 0.001 and ****P < 0.0001. **(F)** Expression of *cIAP2, FADD*, and *RIPK3* in hrHPV + KCs transfected with control siRNA (siControl) or siRNA targeting HPV16 E2 (siE2) stimulated with or without 50 IU/ml IFNγ and 30 ng/ml TNFα for 24 h. Gene expression was normalized against *GAPDH* mRNA levels and standardized against siControl. Similar results were observed in two independent experiments. *P < 0.05, ****P < 0.0001.

Programed cell death knows two major regulatory pathways: caspase-dependent apoptosis and RIP kinases-associated necroptosis (38). To investigate the effects of hrHPV on these pathways, we analyzed the expression of the proteins TRAF2, cIAP1, cIAP2, XIAP, FLIP, CYLD, caspase-8, FADD, RIPK1, RIPK3, and MLKL, involved in formation of the apoptotic or necroptotic signaling complexes (38) at several different time points after IFNγ/TNFα stimulation (Figure 2). We found a consistent difference between KCs and hrHPV-positive KCs with respect to cIAP2, the expression of which was strongly upregulated at both the protein and transcript level by IFNγ/TNFα stimulation in hrHPV-positive KCs only (Figures 2A,D). The cytokine-stimulated expression of TRAF2 seemed to be higher in hrHPV-positive KCs, but this was not confirmed at the transcript level (Figures 2A,D). Analysis of the apoptotic (FLIP, CYLD, caspase-8, FADD) or necroptotic (RIPK1, RIPK3, MLKL) proteins revealed that the levels of FADD were consistently upregulated in hrHPV-positive KCs in response to IFNγ/TNFα treatment. Again, this was not confirmed at the transcript level (Figures 2B,D). Similarly, there was a hint that cFLIP was upregulated at the protein level, but not at the transcript level (Figures 2B,D). In addition, there was no difference between the different KCs in the expression caspase-8 or in the expression of partially cleaved caspase-8 (43 kD) after IFNγ/TNFα treatment (Figure 2B). Fully cleaved caspase-8 (18 kD and 10 kD) was not observed in these blots, unless KCs and hrHPV + KCs were treated with cyclohexamide, which fosters apoptosis by promoting full caspase-8 activation *via* the elimination of c-FLIP (39) (Figure S2 in Supplementary Material). Importantly, RIPK3 was downregulated significantly at the protein level and transcript level by hrHPV (Figures 2C,D). Note that, in hrHPV-positive KCs, the increase in phosphorylated MLKL parallels that of MLKL itself and does not reflect a specific increase in MLKL phosphorylation (Figure 2C). Notably, the expression of many of the proteins in the apoptotic and necroptotic signaling complexes could be increased by either IFNγ or TNFα, but most often the two cytokines synergized in raising the expression of these proteins, in particular RIPK3 (Figure S3 in Supplementary Material). The expression of the other components showed a similar expression in non-infected or hrHPV-positive KCs or varied between cell lines in a non-HPV related manner. To study the role of HPV in the consistently changed components cIAP2, FADD, and RIPK3, the total HPV16 early gene expression was knocked down by introduction of siRNA against HPV16 E2 in hrHPV + KCs (13). This resulted in the reduction of HPV16 gene expression in non- and IFNγ/TNFα-stimulated hrHPV + KC (Figure 2E) as well as in the upregulation of *RIPK3* and unexpectedly also of *cIAP2*, while there was no change in *FADD* (Figure 2F). IFNγ/TNFα stimulation augmented the expression of *cIAP2* and *RIPK3* in KCs when the polycistronic mRNA of HPV16 was knocked down (Figure 2F). These data indicate that only the altered expression of *RIPK3*, but not that of *cIAP2* and *FADD*, can be fully accounted for by an infection of KCs with hrHPV and suggest that hrHPV might impair IFNγ- and TNFα-induced necroptosis.

**Figure 3:**
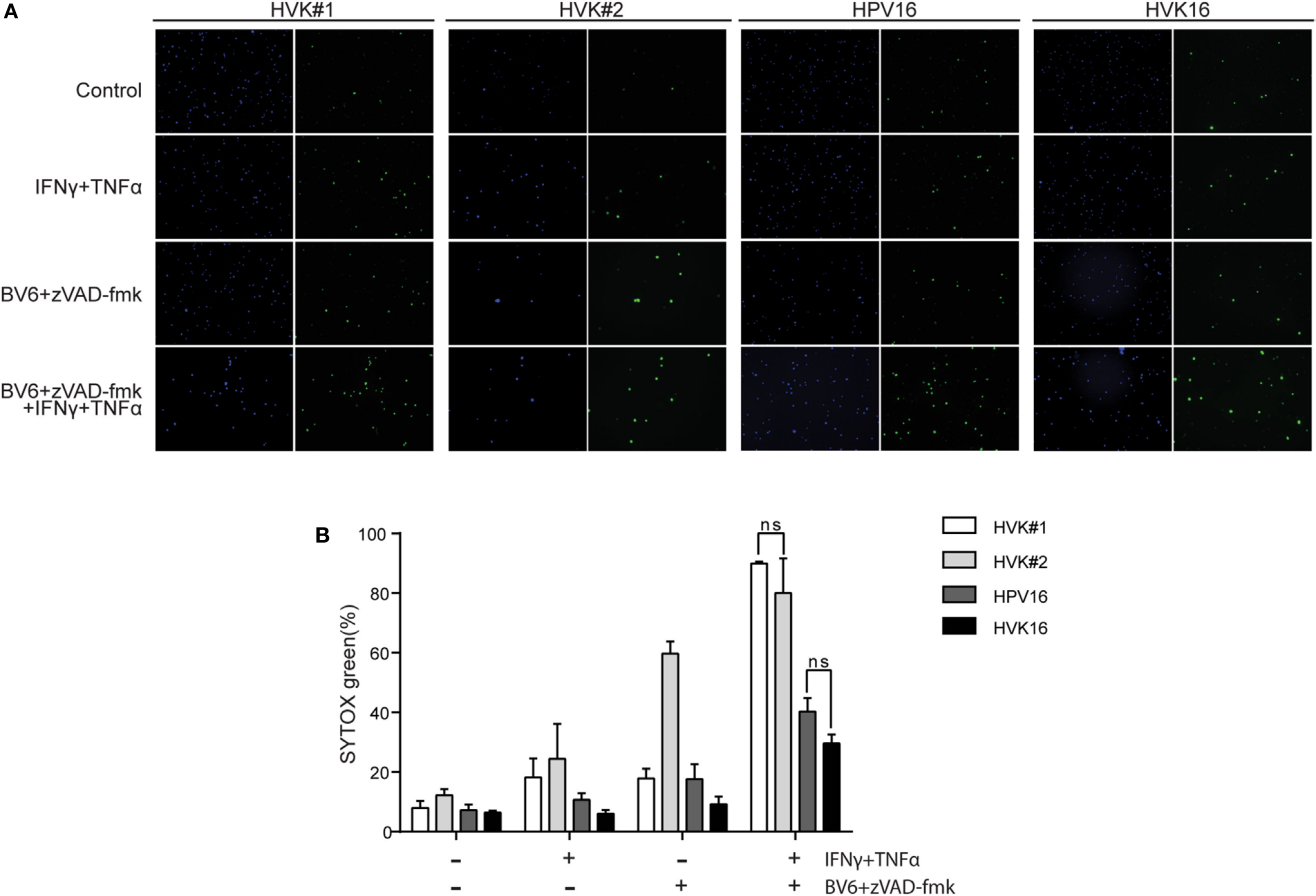
**HPV increases the resistance of KCs to necroptosis**. **(A)** The two independent uninfected KC cultures (HVK#1, HVK#2) and two HPV16 + KC cultures (HVK16, HPV16) were stimulated with 250 IU/ml IFNγ, 250 ng/ml TNFα, 5 μM BV6, and 20 μM zVAD-fmk as indicated for 48 h. All cell nuclei were labeled with DAPI (blue fluorescence). Dead cells were stained using SYTOX green dead cell stain resulting in green fluorescent nuclei of dead cells. **(B)** The number of dead cells among all cells were counted in multiple fields. The percentage of cell death was calculated. *P* values were determined using one-way ANOVA Tukey’s multiple comparisons test. In the group of IFNγ + TNFα + BV6 + z-VAD-fmk, HVK#1 vs. HPV16, *P* < 0.01; HVK#1 vs. HVK16, *P* < 0.001; HVK#2 vs. HPV16, *P* < 0.05; HVK#2 vs. HVK16, *P* < 0.05.

To test this, KCs and hrHPV-positive KCs were stimulated for 48 h with IFNγ and TNFα in the presence of the Smac mimetic BV6 and caspase inhibitor zVAD-fmk in order to promote necroptosis (40), and cell death was analyzed with SYTOX green dead cell stain. While about 80% of the non-infected KCs were killed, a significantly lower percentage, less than 40%, of the hrHPV + KCs died within this 48-h time frame (Figure 3). Consistent with necroptosis, cell death was completely blocked when the RIP kinase 1 inhibitor Nec-1s (41) was present during stimulation (Figure S4 in Supplementary Material). Together, these results show that hrHPV-infected KCs can escape from IFNγ/TNFα-induced necroptosis by downregulating the basal and cytokine-induced expression of RIPK3.

### RIPK3 Downregulation and Resistance to Necroptosis Involves Histone Methyltransferases Overexpressed in hrHPV-Infected KCs

**Figure 4:**
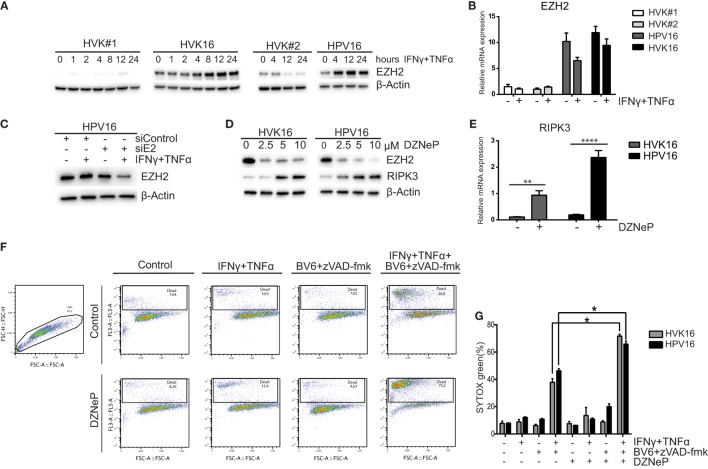
**High-risk HPV-infected KCs overexpress EZH2 and downregulates RIPK3 and to resist necroptosis**. **(A)** Two independent uninfected KC cultures (HVK#1, HVK#2) and two HPV16 + KC cultures (HVK16, HPV16) were stimulated with 50 IU/ml IFNγ and 30 ng/ml TNFα for the indicated times following the protein levels of EZH2 as detected by Western blotting (WB) in whole cell extracts. β-actin served as loading control. **(B)** Two independent uninfected KC cultures (HVK#1, HVK#2) and two HPV16 + KC cultures (HVK16, HPV16) were stimulated with 50 IU/ml IFNγ and 30 ng/ml TNFα for 24 h after which the expression levels of *EZH2* was determined by RT-qPCR. Gene expression was normalized against *GAPDH* mRNA levels and standardized against the non-stimulated uninfected KC culture HVK#1. **(C)** The protein expression level of EZH2 was analyzed in HPV16 + KCs (HPV16) transfected with control siRNA (siControl) or siRNA targeting HPV16 E2 (siE2) and stimulated with or without 50 IU/ml IFNγ and 30 ng/ml TNFα for 24 h, using Western blotting (WB) in whole cell extracts. β-actin served as loading control. **(D)** The protein expression levels of EZH2 and RIPK3 were analyzed in the two independent hrHPV + KCs (HVK16, HPV16) 72 h after pharmacological depletion of EZH2 by increasing doses of 3-deazaneplanocin (DZNeP). **(E)**. The gene expression level of *RIPK3* in 10 μM DZNeP-treated HVK16 and HPV16 hrHPV + KCs after 72 h. Gene expression was normalized against *GAPDH* mRNA levels and standardized against the non-treated HVK16. **P < 0.01, ****P < 0.0001. **(F,G)** Two hrHPV + KCs (HVK16, HPV16) were treated with or without 10 μM DZNeP. After 24 h, the cells were treated with 250 IU/ml IFNγ, 250 ng/ml TNFα, 5 μM BV6, 20 μM zVAD-fmk, and/or 20 μM Nec-1s as indicated for 48 h. The percentage of dead cells, indicated by the cells stained positive by SYTOX green dead cell stain, was measured by flow cytometry. **(F)** Example of analysis by flow cytometry. **(G)** The percentage of cell death measured for each treatment was plotted for both hrHPV + KC cultures. **P < 0.05.

In many cancer cell lines, the expression of RIPK3 is lost due to genomic methylation near its transcriptional start site (42). Recently, it was reported that the methyltransferase EZH2 was overexpressed in HPV16 E6- and E7-transformed KCs (43). Since EZH2 is a core component of polycomb-repressive complex 2 (PRC2) and plays a role in promoter-targeted transcriptional repression (44), we hypothesized that EZH2 may also be involved in repressing the expression of *RIPK3*. Western blot analysis and RT-qPCR of primary KCs and hrHPV-infected KCs revealed that both gene and protein expression of EZH2 was higher at the basal level and after IFNγ/TNFα stimulation in hrHPV + KCs (Figures 4A,B). As expected (44), hrHPV-infected KCs display a higher methylation of H3K27 at the basal level (Figure S5A in Supplementary Material). Knockdown of the polycistronic mRNA of HPV16 in hrHPV16-positive KCs resulted in lower EZH2 protein levels indicating that EZH2 overexpression was induced by hrHPV in KCs (Figure 4C). This effect was even more pronounced after IFNγ/TNFα stimulation fitting with the observation that also the expression of the viral genes was further downregulated (Figure 2E). The use of 3-deazaneplanocin A (DZNep), an inhibitor of *S*-adenosylmethionine-dependent methyltransferase, is known to effectively deplete cellular levels of the PRC2 components, including EZH2 (45). Indeed, treatment of the hrHPV + KCs with DZNep resulted in a dose-dependent decrease in EZH2 protein levels (Figure 4D), decreased of H3K27 methylation (Figure S5B in Supplementary Material), and the concomitant increase in RIPK3 at the protein and gene expression level (Figures 4D,E). Treatment of hrHPV + KCs with the catalytic EZH2 inhibitor GSK503 did not have a clear effect on the expression of RIPK3 (Figure S5C in Supplementary Material), suggesting that the hrHPV-induced overexpression of EZH2 is indirectly responsible for suppressing RIPK3-mediated necroptosis. It should be noted that DZNep has been reported to function as a global histone methylation inhibitor (46). We, therefore, analyzed the expression of other methyltransferases and found that, in addition to EZH2, eight other methyltransferases were expressed at a significantly higher level in hrHPV + KCs (Figures S5D,E in Supplementary Material). Potentially, these methyltransferase may also play a role in downregulating the expression of RIPK3. To test if methyltransferases where involved in suppressing RIPK3-mediated necroptosis, KCs and hrHPV + KCs again were stimulated with IFNγ/TNFα, BV6, and zVAD-fmk, but, now, either in the absence or presence of DZNep. Cell death was determined both by flow cytometry in order to analyze larger numbers of cells (Figure 4F) and by immunofluorescence in cell cultures (Figure S6 in Supplementary Material). Clearly, the presence of DZNep increased the BV6/zVAD-fmk/IFNγ/TNFα-induced percentage of dead cells. Cells died by necroptosis, since cell death could be blocked by Nec-1s (Figures 4F,G; Figure S6 in Supplementary Material).

Thus, by upregulating the expression of histone methyltransferases, thereby effectively decreasing the basal levels of RIPK3, hrHPV increases the immune resistance of KCs to IFNγ/TNFα-stimulated necroptotic cell death.

### The Antiproliferative Effects of IFNγ in KCs Are Counteracted by hrHPV through Downregulation of Interferon-Induced Transmembrane Protein 1

**Figure 5:**
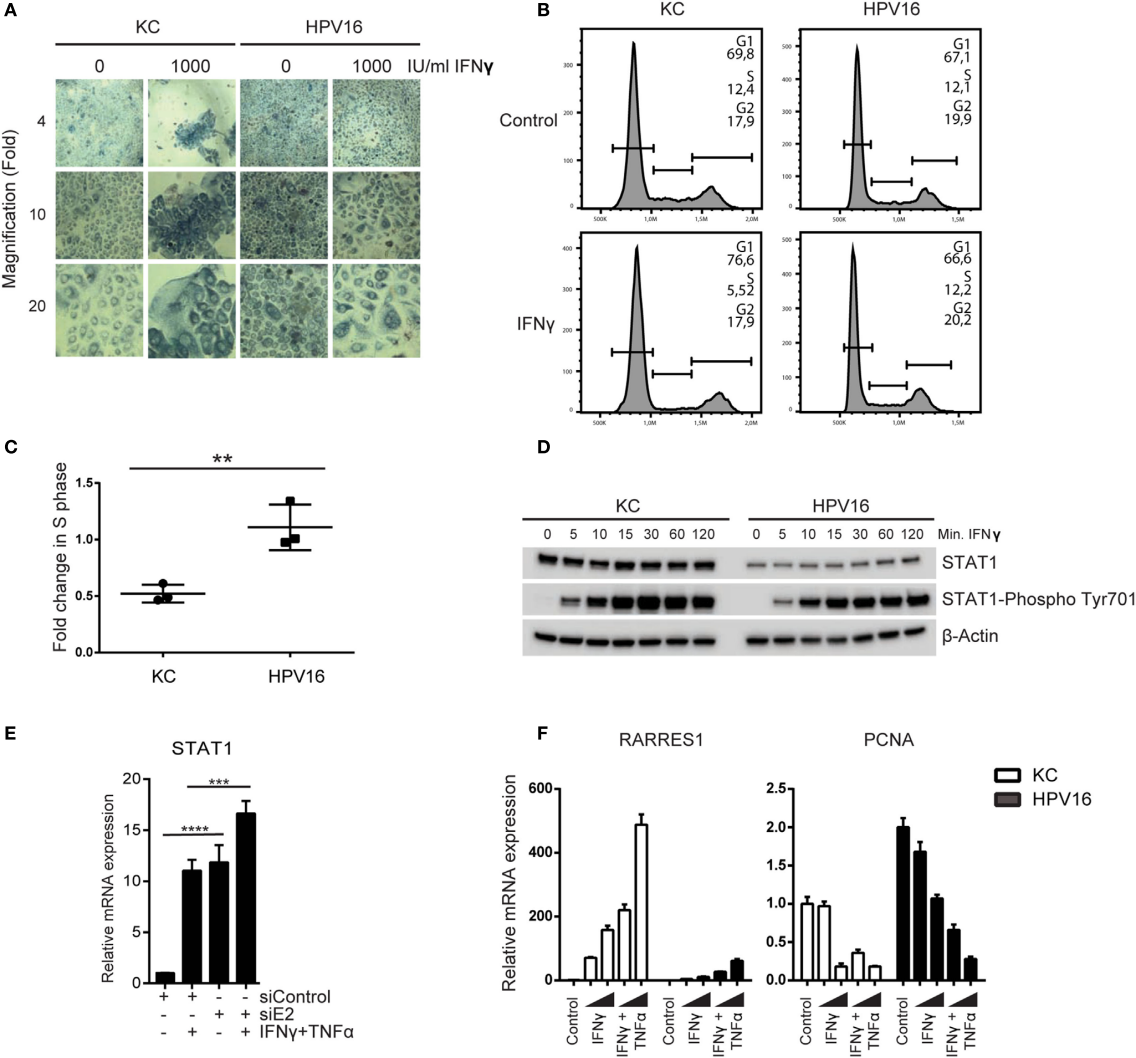
**High-risk HPV resists the antiproliferative effects of IFNγ**. **(A)** Undifferentiated KCs (HVK#1) and HPV16 + KCs (HVK16) were treated with indicated doses of IFNγ for 72 h after which cell confluency was monitored by phase-contrast microscopy as a measure of proliferation. Microscopy pictures (4×, 10×, and 20× magnifications). **(B,C)** The proliferation of undifferentiated KC (HVK#2) and HPV16 + KC (HPV16) treated with 250 IU/ml IFNγ for 48 h was analyzed by examination of the proportion of DNA that was present in the various phases (G1, S, G2/M) of cell growth using PI staining and flow cytometry. **(B)** Example of flow cytometric analysis. The DNA content of 10,000 cells was analyzed. **(C)** The fold-change in the percentage of cells in the S-phase of treated cells over non-treated cells of *n* = 3 experiments is shown. **P < 0.01. **(D)** The protein levels of STAT1 and phosphorylated STAT1 at Tyr701 protein levels in undifferentiated KCs and HPV16 + KCs harvested at indicated time points after stimulation with 50 IU/ml IFNγ as measured by WB is shown. β-actin served as loading control. **(E)** Expression of *STAT1* in HPV16 + KCs transfected with control siRNA (siControl) or siRNA targeting HPV16 E2 (siE2) stimulated with or without 50 IU/ml IFNγ and 30 ng/ml TNFα for 24 h. Gene expression was normalized against *GAPDH* mRNA levels and fold-change over non-stimulated siControl was calculated. ****P < 0.0001, ***P < 0.001. **(F)** Gene expression analysis of *RARRES1* and *PCNA* in undifferentiated KCs and HPV16 + KCs treated with 50 IU/ml IFNγ and 30 ng/ml TNFα for 24 h. Gene expression was normalized against *GAPDH* mRNA levels and fold-change over control was calculated.

Our initial analyses suggested that hrHPV-infected KCs resisted immune-controlled cell growth not only *via* impairment of necroptosis but also by interfering with regulation of proliferation, exemplified by a lower increase in *RARRES1* and downregulation of *PCNA* (Figure 1). Indeed, stimulation of non-infected KCs and hrHPV + KCs with a high dose of IFNγ affects the growth of non-infected KCs but not hrHPV + KCs (Figure 5A). Furthermore, analysis of the cell cycle using flow cytometry and propidium iodide DNA staining, showed that treatment of KCs with IFNγ caused a 50% reduction in the *S*-phase while it hardly affected hrHPV + KCs (Figures 5B,C).

IFNγ signaling requires STAT1 and it has been reported that HPV can lower *STAT1* expression and protein levels in KCs (4–7), potentially explaining the resistance to IFNγ treatment. Indeed, hrHPV-positive KCs displayed a lower expression of STAT1 at the protein level (Figure 5D; Figure S7 in Supplementary Material), and this was due to the presence of hrHPV as knockdown of the polycistronic viral mRNA resulted in a higher *STAT1* expression (Figure 5E). Notably, cytokine stimulation also upregulated *STAT1* expression (Figure 5E), and IFNγ stimulation resulted in high levels of phosphorylated STAT1 in hrHPV + KCs (Figure 5D), indicating that HPV may repress the basal levels of STAT1 but it does not overtly interfere with STAT1 signaling *per se*. This also explains why IFNγ, especially at higher concentrations and in combination with TNFα, is able to stimulate the expression of the antiproliferative gene *RARRES1* and downregulation of the proliferative gene *PCNA* (Figure 5F), albeit that the combinatory effects of IFNγ and TNFα were more pronounced on non-infected KCs (Figure 5F).

**Figure 6:**
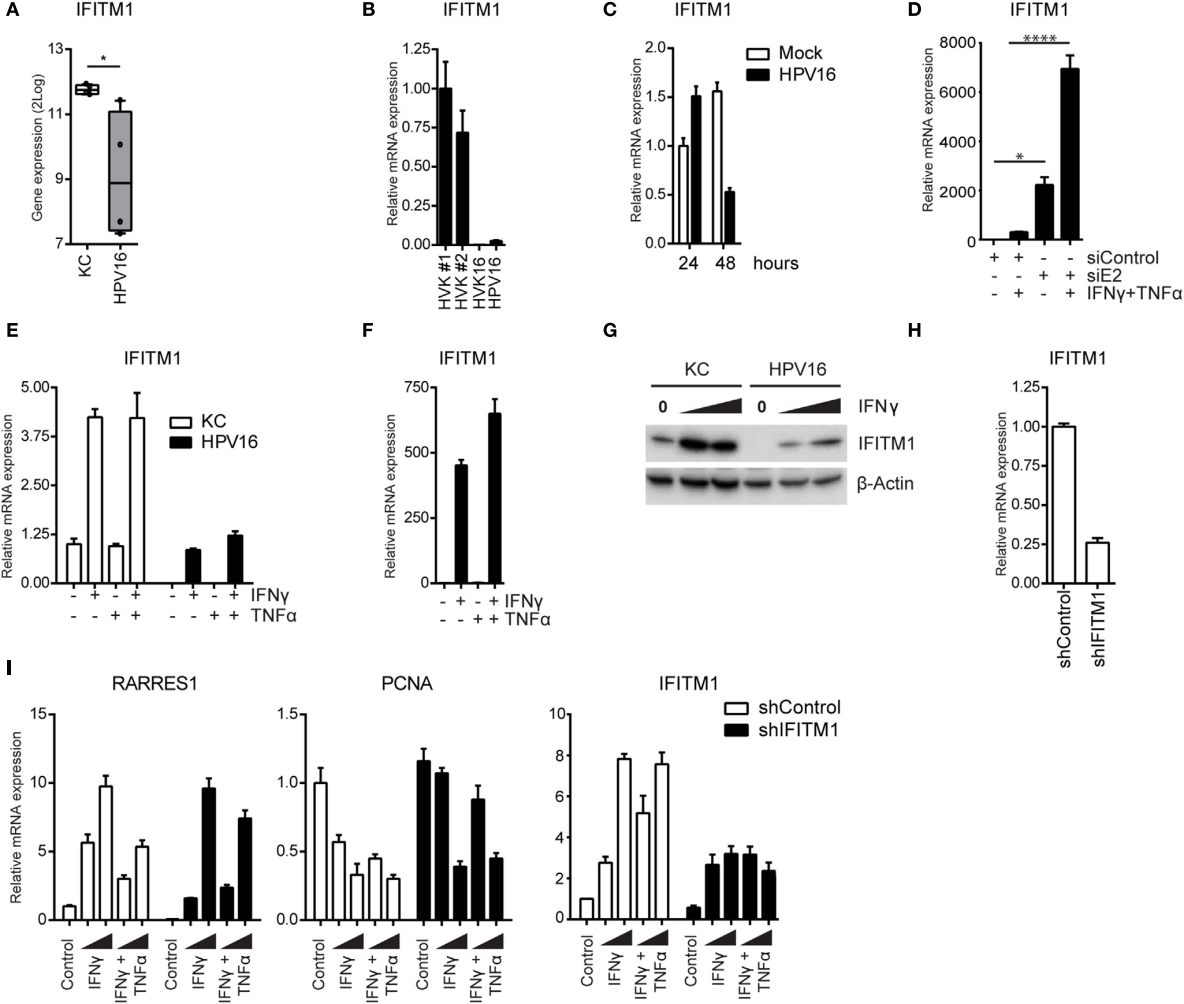
**High-risk HPV-infected KCs downregulate IFITM1 to suppress the expression of *RARRES1* and to maintain proliferation**. **(A)** Microarray gene expression values for *IFITM1* in four independent uninfected KCs and four independent hrHPV + KCs represented in a box plot. *P < 0.05. **(B)** The expression level of *IFITM1* in HVK#1, HVK#2, HVK16, and HPV16 determined by RT-qPCR. Gene expression was normalized against *GAPDH* mRNA levels. **(C)** The expression level of *IFITM1* in KCs infected with mock or native HPV16 virions for 1 or 2 days, respectively. **(D)** Expression of *IFITM1* in HPV16 + KCs transfected with control siRNA (siControl) or siRNA targeting HPV16 E2 (siE2) stimulated with or without 50 IU/ml IFNγ and 50 ng/ml TNFα for 24 h. Gene expression was normalized against *GAPDH* mRNA levels and fold-change over non-stimulated siControl was calculated. *P < 0.05, ****P < 0.0001. **(E, F)** The expression of *IFITM1* in undifferentiated KCs and HPV16 + KCs stimulated with 50 IU/ml IFNγ and/or 50 ng/ml TNFα for 24 h. Gene expression was normalized against *GAPDH* mRNA levels and fold-changes over **(E)** control-stimulated undifferentiated KCs or over **(F)** control-stimulated HPV16 + KCs were calculated and depicted. **(G)** IFITM1 protein levels in KC and HPV16 + KC stimulated with 0, 100, or 1000 IU/ml IFNγ, as measured by WB. β-actin served as loading control. **(H)** *IFITM1* expression in control and IFITM1 knockdown uninfected KCs as measured by RT-qPCR. Gene expression was normalized against *GAPDH* mRNA levels and fold-change over siControl was calculated. **(I)** Control and *IFITM1* knockdown KCs were stimulated with 50 IU/ml IFNγ and/or 50 ng/ml TNFα for 24 h before the expression of *RARRES, PCNA*, and *IFITM1* was measured by RT-qPCR. Gene expression was normalized against *GAPDH* mRNA levels and fold-change over non-stimulated shControl was calculated.

Interferon-induced transmembrane protein 1 (IFITM1) plays an essential role in the antiproliferative action of IFNγ (47), making it a potential target for hrHPV. Re-analysis of the data from one of our earlier validated microarrays, in which the basal expression of genes measured in different uninfected and hrHPV-infected KCs was compared in the absence of IFNγ stimulation (31), showed that *IFITM1* expression is downregulated in HPV-positive KCs (Figure 6A). This was confirmed by RT-qPCR (Figure 6B). To show that the expression of IFITM1 was genuinely altered by the presence of hrHPV in KCs, undifferentiated KCs were infected with native HPV16 virions resulting in a reduced expression of *IFITM1* (Figure 6C). Reciprocally, the knockdown of total HPV16 early gene expression in hrHPV + KCs resulted in the upregulation of *IFITM1* (Figure 6D). IFNγ induces *de novo* synthesis of *IFITM1* for which STAT1 is required (48–51). Indeed, IFNγ stimulation of uninfected KCs resulted in approximately fourfold increase in *IFITM1* after 24 h (Figure 6E). Strikingly, IFNγ stimulation of hrHPV + KCs resulted in a much stronger relative increase of IFITM1 levels (Figure 6F), albeit that these levels still remained lower than those measured in uninfected KCs (Figure 6E). IFITM1 protein levels in IFNγ-stimulated KCs and hrHPV + KCs confirmed the gene expression data (Figure 6G). These data indicated that hrHPV predominantly regulates the expression of *IFITM1* at the basal level but less at the level of IFNγ-mediated induction of *IFITM1* gene expression. Interestingly, the hrHPV + KCs with the highest basal IFITM1 protein expression also showed the highest STAT1 levels (Figure S7 in Supplementary Material). TNFα did not influence *IFITM1* expression (Figures 6E,F).

To study the effects of IFITM1 on KC proliferation in a setting where all additional influences of HPV are ruled out (52, 53), IFITM1 was knocked down in uninfected KCs (Figure 6H). The KCs were stimulated with IFNγ or IFNγ/TNFα. The basal level of *RARRES1* was lower in IFITM1 knocked-down KCs, and its IFNγ-induced expression was clearly affected when KCs were stimulated with a low concentration of IFNγ (Figure 6I). A higher concentration of IFNγ overcame the effect of IFITM1 knockdown on the expression of *RARRES1*. In addition, IFITM1-knockdown KCs displayed a less strong downregulation of *PCNA* upon IFNγ stimulation (Figure 6I). The less pronounced effects on *RARRES1* and *PCNA* at the higher IFNγ concentrations used was probably due to the fact that upon IFNγ stimulation still an increase in *IFITM1* could be observed in IFITM1 knockdown KCs (Figure 6I). In addition, control shRNA-transduced KCs were less resistant than IFITM1 knockdown KCs to the antiproliferative effects of IFNγ and the combination of IFNγ and TNFα when cell confluency was monitored by phase-contrast microscopy or cell number was quantitated by a DNA-based proliferation assay (Figures S8A,B in Supplementary Material). Thus, HPV is able to resist IFNγ-mediated arrest of proliferation by lowering the basal levels of IFITM1.

## Discussion

In most cases, the immune system succeeds in controlling hrHPV infections but this process takes time and requires the presence of strong IFNγ and TNFα-associated HPV-specific T cell responses (16). Here, we show that hrHPV-infected KCs resist the immune system by interfering with the regulation of intracellular growth and cell death programs of infected cells. Under normal circumstances, these would be activated in response to the effector molecules of the adaptive immune system and function as a host defense mechanism to control viral spread (54). Using a unique *in vitro* model, we showed that hrHPV infection renders KCs resistant to IFNγ/TNFα-induced necroptosis and arrest of cell growth. HrHPV infection is associated with the upregulation of nine methyltransferases, including EZH2, and downregulation of the expression of RIPK3, which results in an impaired induction of necroptosis by IFNγ/TNFα stimulation. Use of DZNep, a global inhibitor of methyltransferases and a pharmacological compound that depletes EZH2 (45, 46), restored the expression of RIPK3 and the sensitivity of hrHPV-infected KCs to IFNγ/TNFα-mediated necroptosis. Use of the catalytic EZH2 inhibitor GSK503 did not restore RIPK3 expression, suggesting that either EZH2 is indirectly responsible for suppressing RIPK3-mediated necroptosis or that one or more of the other overexpressed methyltransferases are involved in the downregulation of RIPK3. Furthermore, hrHPV effectively downregulated the basal expression of the negative regulator of cell growth *IFITM1*, resulting in an impaired IFNγ-mediated increase in the expression of the antiproliferative *RARRES1* gene and decrease of the proliferative gene *PCNA* as well as impaired arrest of cells in the S-phase. Knockdown of *IFITM1* with siRNA in normal KCs recapitulated the effects on *RARRES1* and *PCNA* expression and cell proliferation observed in hrHPV + KCs.

Apoptosis and necroptosis play an important role in controlling viral infections (54). We did not observe any HPV-induced differences in the expression of caspase-8, FLIP, or FADD, nor did we observe differences in cleavage of caspase-8 upon stimulation with IFNγ and TNFα. Notably, stimulation with IFNγ and TNFα did not lead to activation of caspase-8, reflected by the absence of fully cleaved caspase-8 in undifferentiated normal or hrHPV-positive KCs. However, upon differentiation, KCs become sensitive to Fas- and caspase-8-mediated apoptosis following stimulation with IFNγ and TNFα (29, 30). In contrast, stimulation of undifferentiated KCs did result in necroptosis following an increase of RIPK3, most notably when both IFNγ and TNFα were used. The induction of necroptosis has shown to be important for the control of vaccinia virus (55) and herpes simplex virus type 1 (56). Consequently, viruses have developed strategies to resist this immune control mechanism. The murine cytomegalovirus expresses the M45-encoded inhibitor of RIP activation (vIRA) that targets RIPK3 and disrupts RIPK1–RIPK3 interactions characteristic for necroptosis (57). Rather than by interrupting necroptosis, hrHPV prevents the formation of the necrosome by reducing the levels of RIPK3 *via* the upregulation of histone methyltransferases.

The production of new HPV particles requires proliferation and differentiation of infected basal KCs. An arrest in cell proliferation, therefore, is an effective means to control viral infection. IFITM1 plays an essential role in the antiproliferative action of IFNγ (47), thus lowering its expression – as observed in hrHPV-infected KCs – may allow viral escape. Indeed, hepatitis C virus was found to decrease the expression of *IFITM1 via* the upregulation of mIR-130a in order to sustain its replication (58). We showed that the basal expression of *IFITM1* is downregulated in hrHPV + KCs, but its downstream partner *RARRES1* is not. This might be explained by the fact that the basal expression of *RARRES1* in uninfected KCs is already low. The IFNγ-induced increase in expression of *IFITM1* and *RARRES1* requires signaling *via* the IFNGR1 and STAT1. Overexpression of EZH2 has been reported to suppress the expression of IFNGR1 in MYC- transformed cells but not phosphatidylinositol 3-kinase (PI3K)-transformed cells, despite the fact that in both types of transformed cells EZH2 was overexpressed (59). Notwithstanding the ectopic expression of EZH2 in hrHPV-positive KCs, there were no specific differences in the expression of IFNGR1 between non-infected and hrHPV-positive cells. This is in line with the observation that the PI3K pathway is a major target for the hrHPV proteins (59, 60) and suggests that the overexpression of EZH2 does not play a role in the escape of hrHPV-positive cells at this level. It was previously reported that the HPV early proteins E6 and E7 downregulate the expression of STAT1 (4–6). Our data confirm that infection with hrHPV decreases basal STAT1 protein levels in KCs but also show that hrHPV does not interfere with IFNγ-induced STAT1 activation *per se*, as reflected by STAT1 phosphorylation and increase in *RARRES1* and *IFITM1* expression. Still, as total STAT1 levels are lower in hrHPV + KCs, the reduced total amount of activated STAT1 may explain why in hrHPV + KCs the increase in *RARRES1* and *IFITM1* expression does not reach the levels observed in uninfected KCs. This is also demonstrated by the data showing that the effect of *IFITM1* knockdown on proliferation of uninfected KCs is not similar to what hrHPV has on KCs. While the effect of IFITM1 in uninfected KCs is apparent and antiproliferative, indicated by the retained expression of *PCNA* and *RARRES1* in KCs stimulated with a low dose of IFNγ when *IFITM1* was knocked down, clearly the downregulation of STAT1 as well as the positive growth signals as delivered by hrHPV (52, 53) are missing in these cells. Hence, differences in IFNγ-stimulated arrest of proliferation are less noticeable. This shows that, whereas the decreased basal level of *IFITM1* is already providing resistance to the IFNγ-stimulated arrest of proliferation, the downregulation of STAT1 is likely to exaggerate this effect. Mechanistically, IFITM1 inhibits the phosphorylation of ERK and thereby regulates mitogen-activated protein (MAP) kinase signaling. Furthermore, IFITM1 mediates the de-phosphorylation of p53 at Thr55, resulting in increased p53 stability and transcriptional activity and the upregulated expression of p21. Consequently, there is an arrest in cell cycle progression and hence a stop in proliferation (47). This was also observed in this study and reflected by the retained *PCNA* expression when *IFITM1* was knocked down in low dose IFNγ-stimulated KCs.

In conclusion, hrHPV controls proliferation by regulating the expression of (anti-)proliferative genes *via* STAT1 and IFITM1 and resists the induction of necroptotic cell death by downregulation of RIPK3 expression. This allows infected KCs to partly resist immune pressure by IFNγ/TNFα and explains how hrHPV can partially evade the effector mechanisms of the immune system, which may ultimately lead to progression of hrHPV-induced lesions.

## Author Contributions

Design of study: JB and SB. Performed experiments: WM, BT, and RG. Delivered materials: EE and CM. Analyzed data: WM, BT, RG, and JB. Interpreted data: WM, BT, RG, EE, CM, CJMM, JB, and SB. Writing: WM, BT, CM, CJMM, JB, and SvdB.

## Conflict of Interest Statement

The authors declare that the research was conducted in the absence of any commercial or financial relationships that could be construed as a potential conflict of interest.
