# Supplementary figures and images for: Human Papillomavirus Downregulates the Expression of IFITM1 and RIPK3 to Escape from IFNγ- and TNFα-Mediated Antiproliferative Effects and Necroptosis
Source: Front Immunol. 2016 Nov 22;7:496. doi: 10.3389/fimmu.2016.00496 (PMC5118436; doi:10.3389/fimmu.2016.00496)

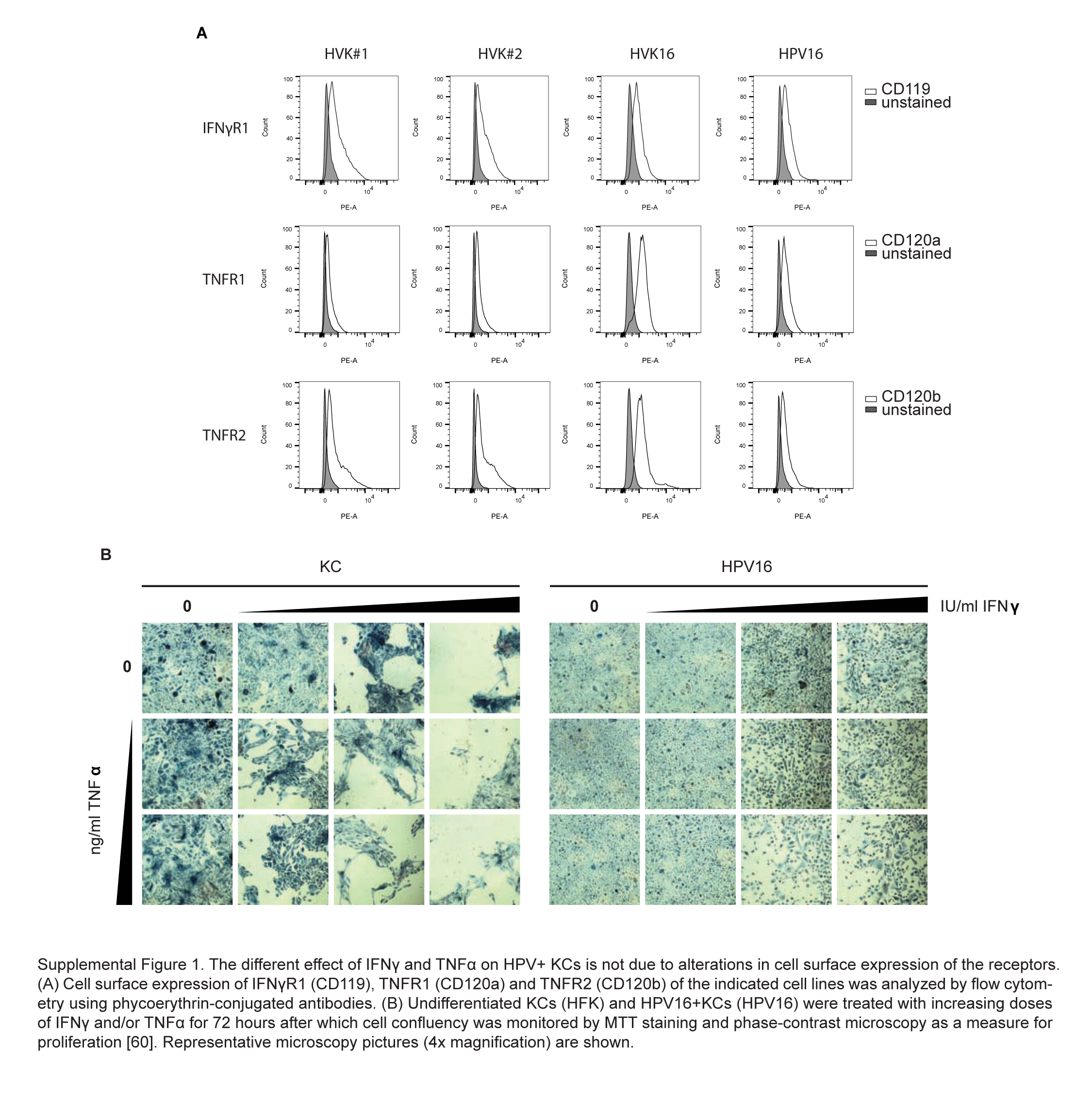

Supplement: Supplementary file 1 [file Image_1.TIF]

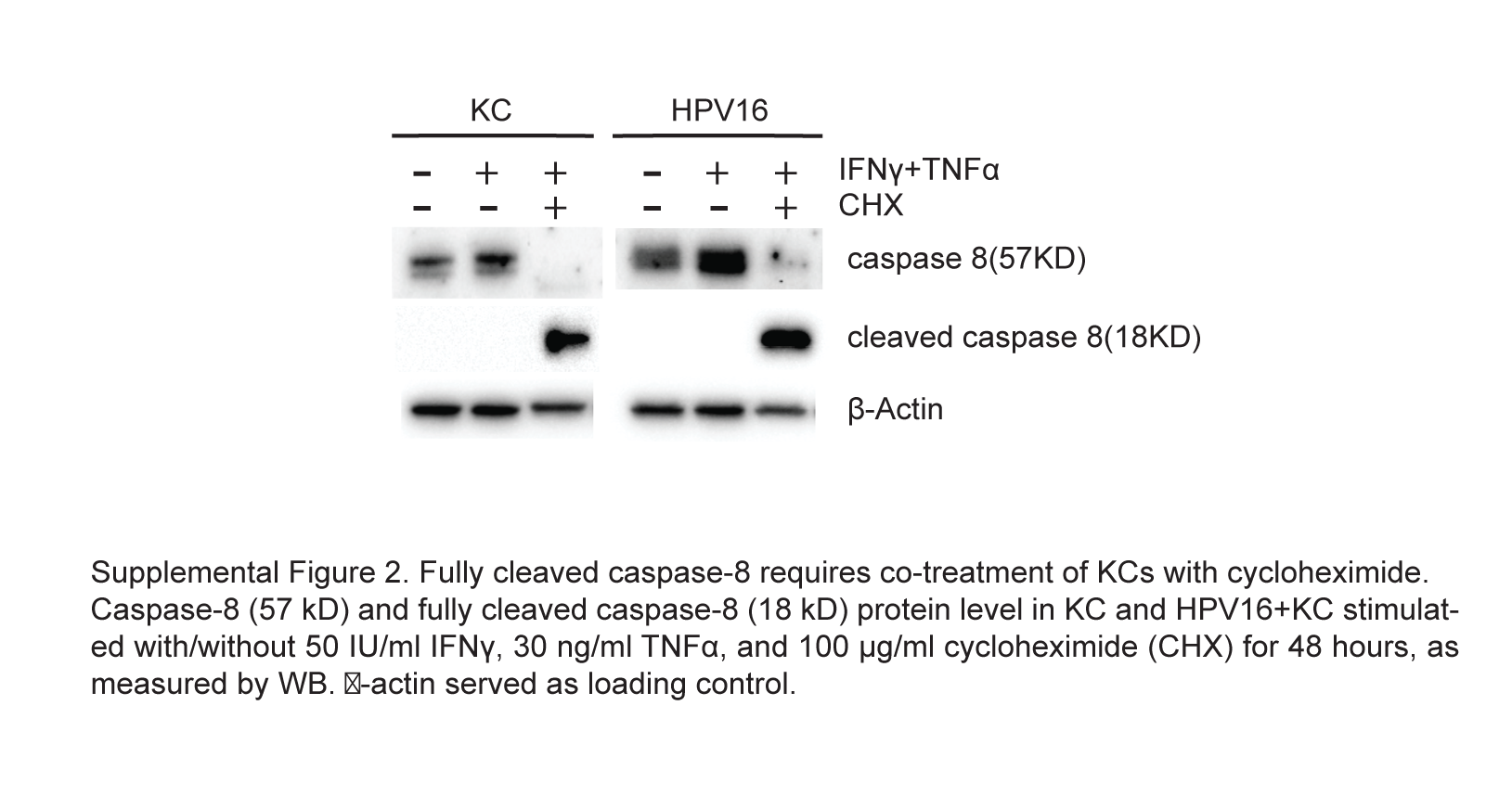

Supplement: Supplementary file 2 [file Image_2.TIF]

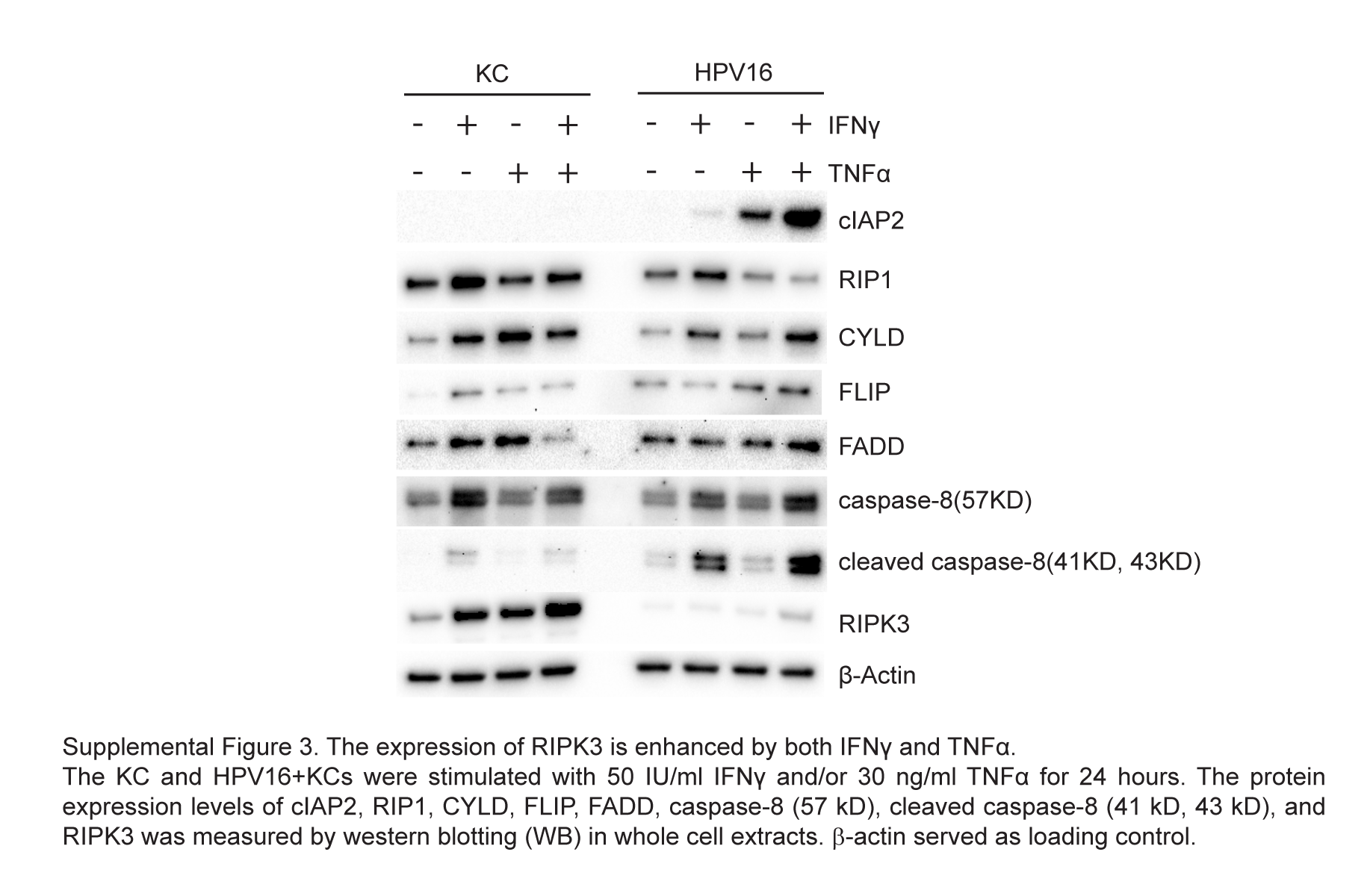

Supplement: Supplementary file 3 [file Image_3.TIF]

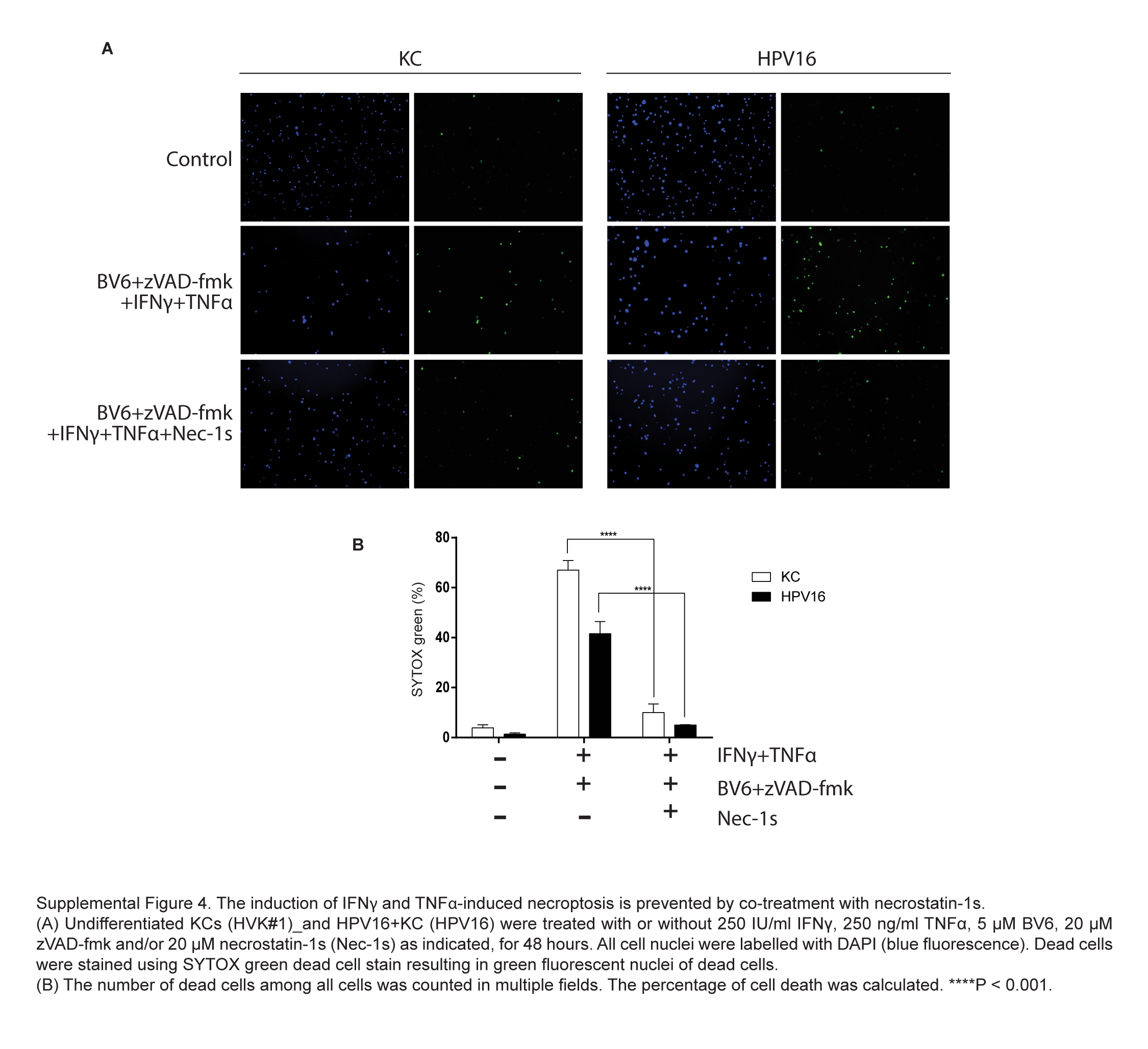

Supplement: Supplementary file 4 [file Image_4.TIF]

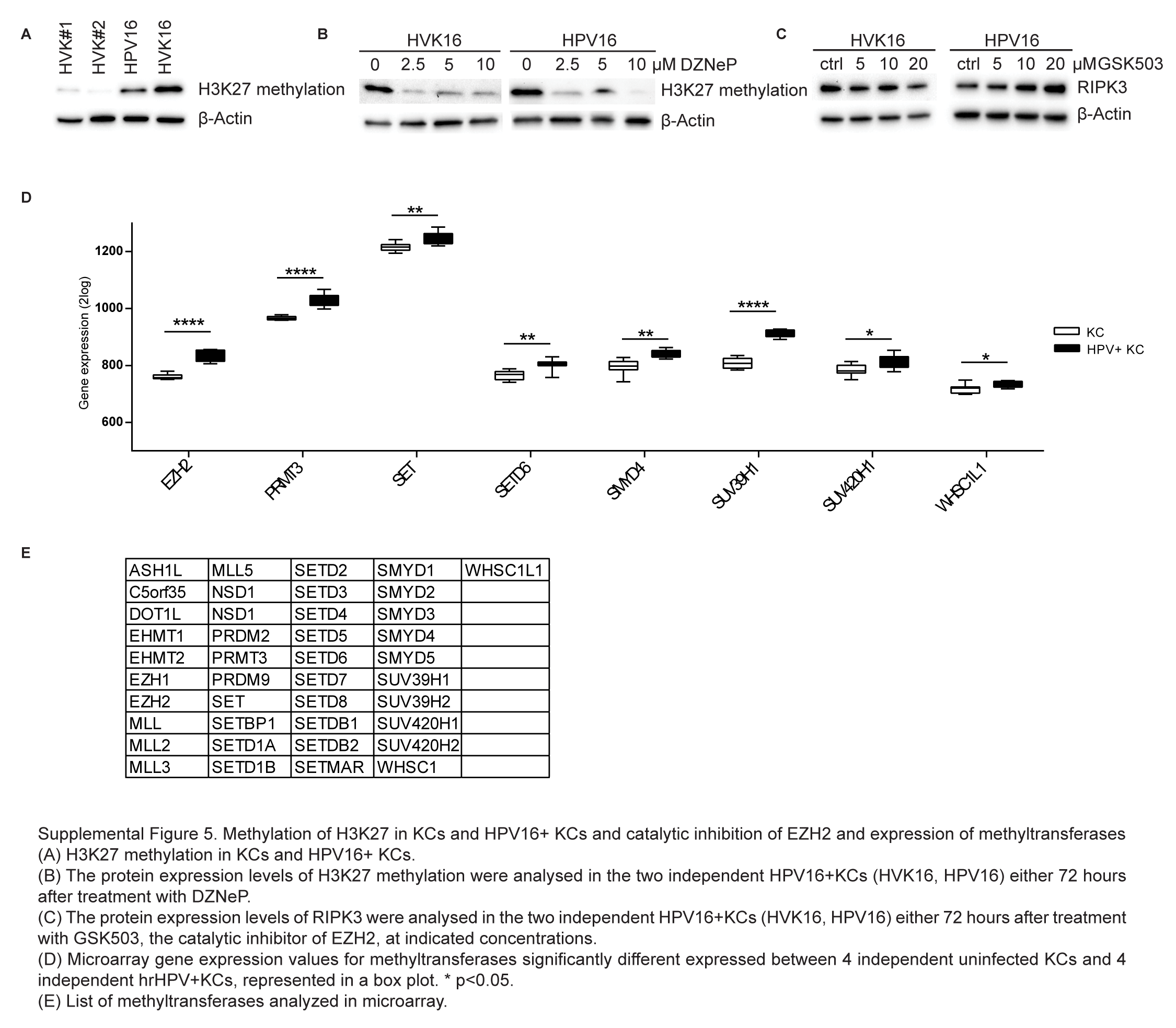

Supplement: Supplementary file 5 [file Image_5.TIF]

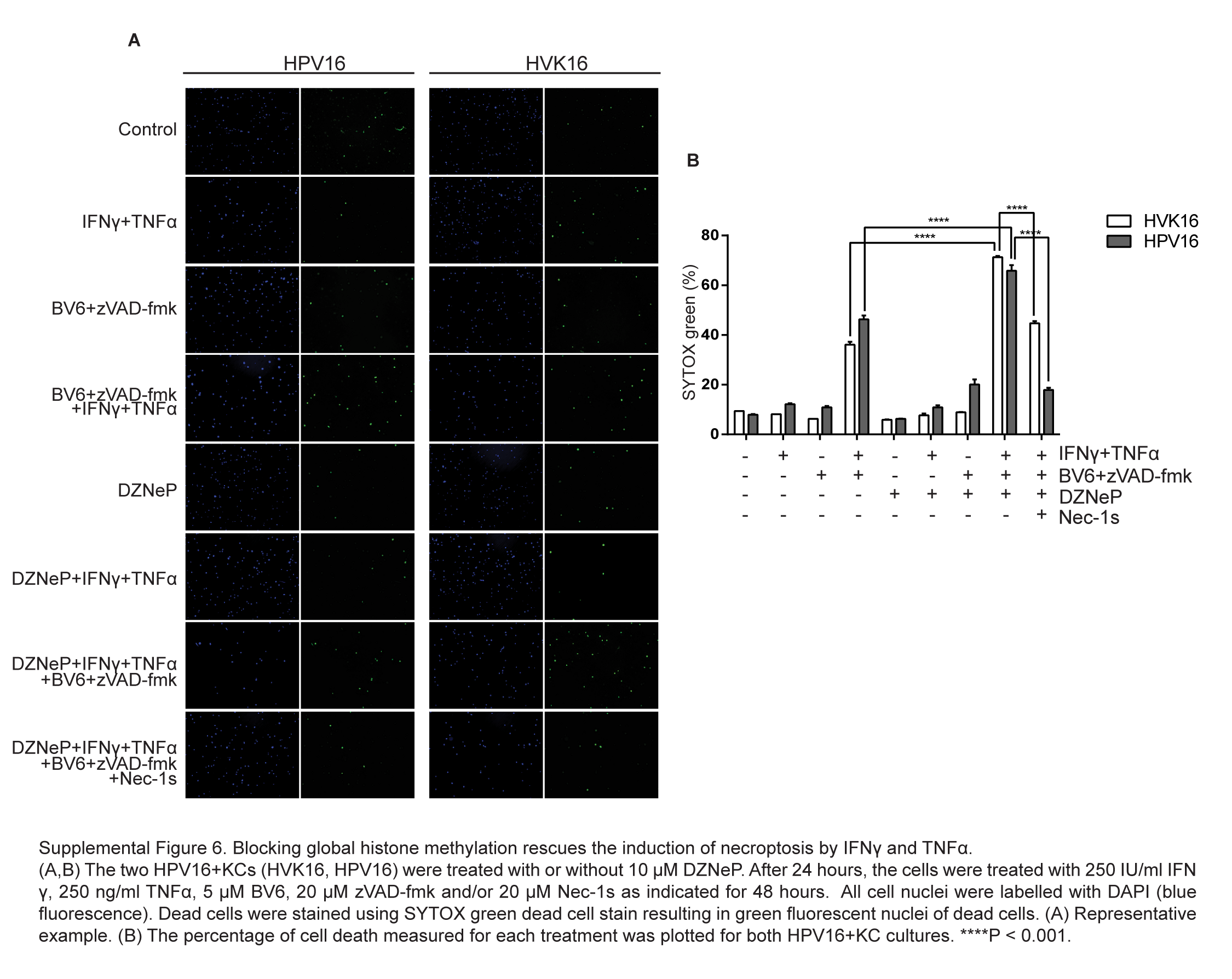

Supplement: Supplementary file 6 [file Image_6.TIF]

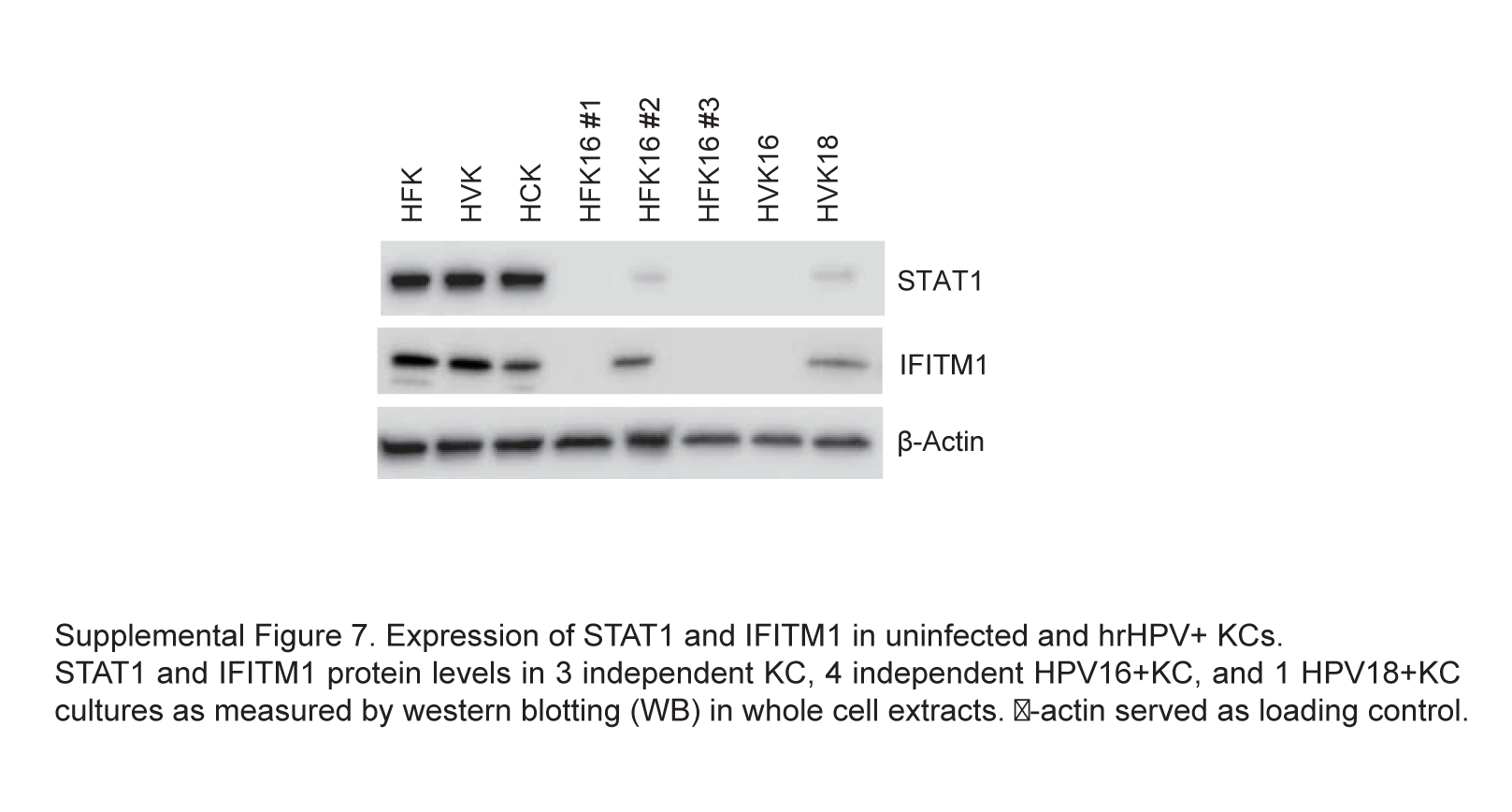

Supplement: Supplementary file 7 [file Image_7.TIF]

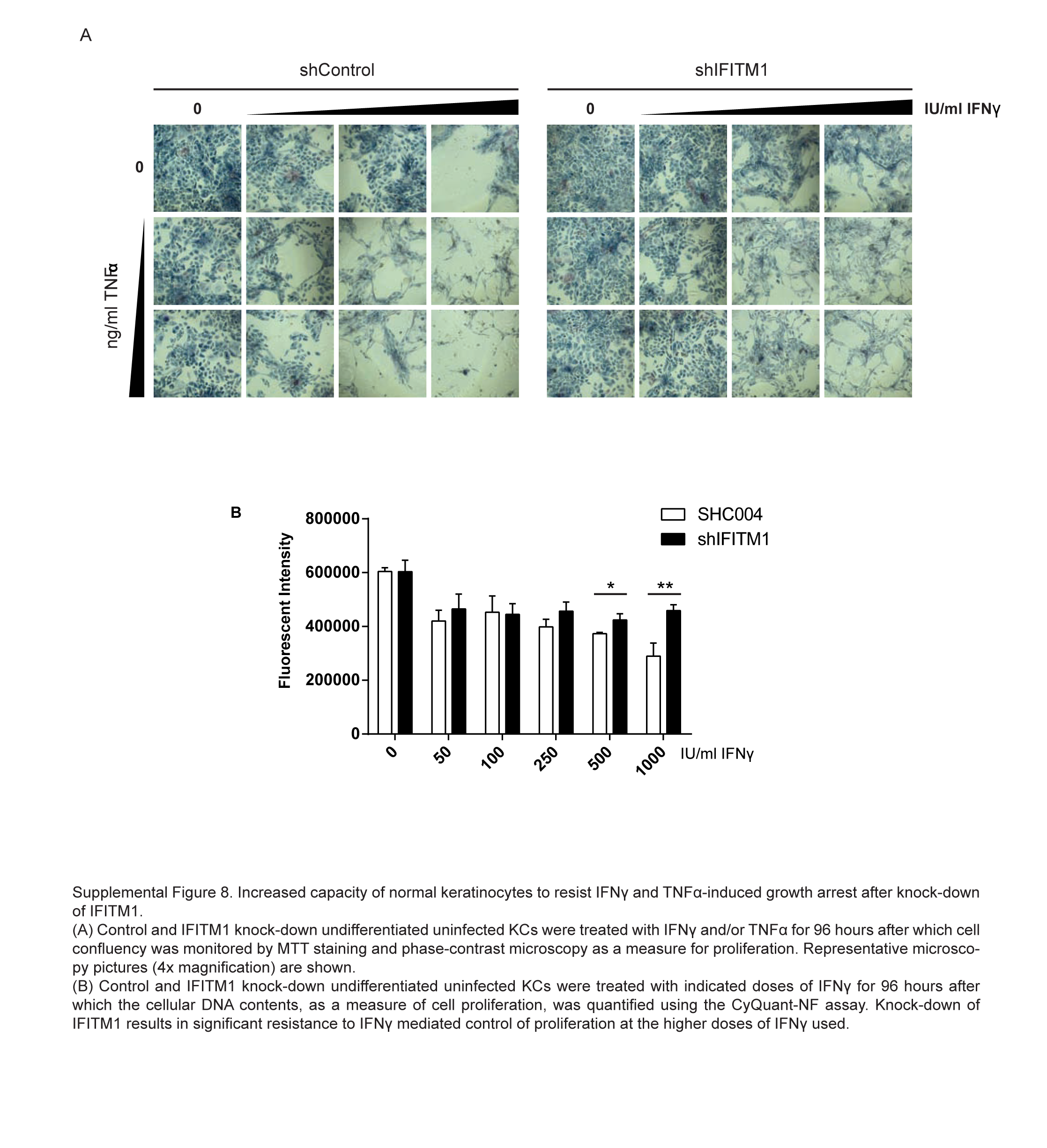

Supplement: Supplementary file 8 [file Image_8.TIF]
